# Contrast-induced nephropathy following angiography and cardiac interventions

**DOI:** 10.1136/heartjnl-2014-306962

**Published:** 2016-02-08

**Authors:** Roger Rear, Robert M Bell, Derek J Hausenloy

**Affiliations:** 1 The Hatter Cardiovascular Institute, University College London, London, UK; 2 The National Institute of Health Research University College London Hospitals Biomedical Research Centre, London, UK; 3 National Heart Research Institute Singapore, National Heart Centre Singapore, Singapore, Singapore; 4 Cardiovascular and Metabolic Disorders Program, Duke-National University of Singapore, Singapore, Singapore

**European Society of Cardiology (ESC) curriculum section and guidelines referenced**

- 2.4 Invasive imaging: cardiac catheterisation and angiography.
- ESC: updated contrast-induced nephropathy (CIN) prevention guidelines 2014.
- European Society of Urogenital Radiology: updated contrast media safety committee guidelines 2011.

**Learning objectives**

- Define CIN and recognise this as a common and serious complication in susceptible patients receiving intravascular contrast media.
- Understand the possible pathological mechanisms underlying CIN.
- Describe the clinical and periprocedural risk factors for CIN and perform a risk assessment for patients receiving contrast media.
- Appreciate the established strategies used to prevent CIN and be aware of novel therapies.
- Recognise the onset of CIN and manage this complication appropriately.

## Introduction

Contrast-induced nephropathy (CIN), also known as contrast-induced acute kidney injury, is an iatrogenic renal injury that follows intravascular administration of radio-opaque contrast media (CM) in susceptible individuals. CIN was first described during the 1950s in case reports of fatal acute renal failure that had occurred following intravenous pyelography in patients with renal disease arising from multiple myeloma.^1^ ^2^ Despite technological advances, CIN remains responsible for a third of all hospital-acquired acute kidney injury (AKI)^3^ ^4^ and affects between 1% and 2% of the general population and up to 50% of high-risk subgroups following coronary angiography (CA) or percutaneous coronary intervention (PCI).^5^

The proliferation of imaging methods and interventional procedures involving administration of intravascular CM in both non-cardiac modalities (eg, vascular CT angiography and interventional vascular angiography) and in established (eg, CA and PCI) and emerging cardiac modalities (eg, CT coronary angiography (CTCA) and transcatheter aortic valve implantation (TAVI)) has significantly increased the number of patients exposed to CM and thus the number at risk of CIN. The widespread adoption of primary PCI for the treatment of acute myocardial infarction (AMI), despite significantly improving cardiovascular outcomes, has increased the incidence of CIN due to the inherent difficulties in rapidly assessing CIN risk, instigating prophylactic measures, attendant haemodynamic compromise and higher contrast volumes, all known risk factors for the development of CIN.^6^ Despite several therapeutic approaches, the rising age and incidence of comorbidity within the broad cohort of cardiac patients receiving CM has ensured that the prevention of CIN remains a significant clinical challenge.^7^

As will be discussed in the following sections, the estimated risk of an individual developing CIN can be calculated using known pre-existent clinical and periprocedural factors, which are consistent with the proposed pathological mechanisms of CIN. Pre-existent stage III chronic kidney disease (CKD), defined as an estimated glomerular filtration rate (eGFR)<60 mL/min/1.73 m^2^ for greater than 3 months, is the most commonly identified risk factor for CIN; however, CIN can occur in the absence of underlying CKD if a number of other risk factors are also present.^5^ Risk scoring systems have been developed from cohort studies^8^ ^9^ that have enabled clinicians to predict the likelihood of CIN occurrence and have allowed targeted use of preventative therapies. The wholly iatrogenic and predictable nature of CIN makes it a particularly well-suited area for ongoing cardiovascular and nephrology research, with focus on pathophysiological mechanisms as well as novel risk assessment, preventative, diagnostic and therapeutic measures.

## Definition and diagnostic criteria of CIN

The generally accepted definition of CIN is a 25% relative increase, or a 0.5 mg/dL (44 µmol/L) absolute increase, in serum creatinine (SCr) within 72 h of contrast exposure, in the absence of an alternative explanation.^10^ Criticisms of this definition include the lack of sensitivity to minor increases in SCr that have been shown to correlate with adverse events,^11^ ^12^ the combination of both relative and absolute SCr changes and the absence of any functional assessment such as changes in urine output, as used in the RIFLE,^13^ AKIN^14^ and KDIGO^15^ classification systems. However, this definition has the advantage of being widely used as the end point in most CIN studies and it correlates well with adverse clinical end points.

An alternative definition proposed by Harjai *et al*^16^ aims to classify CIN according to three grades corresponding to three relative and absolute creatinine rise cut-offs, including a group with only minor rise (<25% or 0.5 mg/dL), which also correlates with long-term adverse outcomes (table 1).

**Table 1:** CIN severity grading system (adapted from Harjai *el al*^16^)

CIN grade	Change in serum creatinine	6 month outcomes
Grade 0	SCr increase <25% and <0.5 mg/dL above baseline	MACE 12.4% Mortality 10.2%
Grade 1	SCr increase ≥25% and <0.5 mg/dL above baseline	MACE 19.4% Mortality 10.4%
Grade 2	SCr increase ≥0.5 mg/dL above baseline	MACE 28.6% Mortality 40.9%

CIN, contrast-induced nephropathy; MACE, major adverse cardiovascular event; SCr, serum creatinine.

A significant problem with SCr is that it is relatively insensitive to the rapid GFR changes seen in AKI, particularly in patients with normal baseline renal function.^17^ Elevations in SCr typically take 2–3 days to reach the current diagnostic threshold following an acute renal insult, thus reducing its usefulness as a marker of AKI. However early and minor incremental changes in SCr may be a useful marker of CIN; a recent clinical trial performed by Ribichini *et al*,^18^ which included 216 at-risk patients undergoing CA, demonstrated that at 12 h a 5% increase in SCr from baseline was a sensitive (75%) and specific (72%) marker of CIN at 48 h and persistent worsening of renal function at 30 days.

Several novel renal biomarkers, including NGAL,^19^ Cystatin C,^20^ urinary Kim-1^21^ and interleukin-18^22^ have been proposed to specifically detect CIN within minutes to hours of the renal insult. Unfortunately, to date, these biomarkers have yet to progress beyond the realms of clinical research; there is a clear clinical need for large trial validation of their use in the early detection and intervention in AKI.

## Adverse outcomes following CIN

CIN is often regarded in clinical practice as a transient event; in up to 80% of cases, SCr levels normalise after approximately 1–3 weeks.^23^ However, CIN is of clinical importance as a number of clinical trials have revealed that it portends a multitude of short-term and long-term adverse events.^24^ After adjusting for comorbidities, observational studies have demonstrated that in-hospital mortality is approximately five times higher in patients who suffer CIN compared with patients receiving CM who do not,^25^ and mortality rates at 1 and 5 years are approximately four times higher,^26^ with some demonstrating a 1-year mortality rates of between 20%^12^ and 38%.^27^ In other observational studies, as many as 20% of those developing CIN suffer persistent worsening renal function after CM exposure,^28^ with renal replacement therapy occurring in between 0.7%^25^ and 7%^27^ of patients with CIN (table 2). As such the additional healthcare costs associated with CIN are thought to be considerable.^12^

**Table 2:** Cardiovascular adverse outcomes following CIN

Adverse event	Outcome: CIN group vs no CIN group
In-hospital mortality	7.1% vs 1.1% (p<0.0000001) McCullough *et al*,^25^ n=1826
1 year mortality	37.7% vs 19.4% (p=0.001) Gruberg *et al*,^27^ n=439
Persistent worsening of renal function (eGFR>25% baseline at 3/12)	18.6% vs 0.9% (p=0.0001) Maioli *et al*,^28^ n=1490
Haemodialysis	0.7% McCullough *et al*^25^ 7% Gruberg *et al*^27^

CIN, contrast-induced nephropathy; CM, contrast media; eGFR, estimated glomerular filtration rate.

However, it is important to recognise that a direct causal relationship between CIN and mortality has not been established in these observational studies. The onset of CIN is more likely to occur in the presence of severe cardiac injury or disease, which alone conveys a poor prognosis. As such CIN may be a marker of adverse cardiovascular outcomes rather than an independent risk factor. A recent meta-analysis by James *et al*,^29^ reviewed 39 observational studies that investigated cardiovascular outcomes in those with CIN and demonstrated an increased risk of mortality, cardiovascular events, renal failure and prolonged hospitalisation. However, it was found that baseline clinical characteristics that simultaneously predispose to both CIN and mortality were strong confounders, especially so in unadjusted studies. Even with appropriate adjustment for comorbidity, the authors recommend that any firm conclusions about causality should be interpreted with caution.

Nonetheless, a number of plausible pathological mechanisms exist that might explain a direct link between CIN and major adverse cardiac events (MACE). In the short term these include acute volume overload, electrolyte disturbance, uraemia or haemodialysis (HD) in cases of severe CIN. Longer term MACE in those who suffer persistent renal injuries may be increased secondary to the cardiovascular risk associated with progressive CKD and its many pathological manifestations, including accelerated atherosclerosis, vascular calcification and left ventricular (LV) hypertrophy.^30^ However, it is less conceivable how minor or transient changes in kidney function might increase MACE risk.

In order to demonstrate a definite causal link between CIN and MACE, large randomised controlled trials (RCTs) are needed to demonstrate that effective CIN prevention strategies are also able to reduce short-term and long-term MACE, specifically using therapies that offer no additional cardiovascular risk reduction benefits. Given the similarities that exist between renal and cardiovascular disease and their respective treatments, this is unlikely to be feasible. In view of the many complex confounders associated with MACE, CIN clinical trials that focus exclusively on clinically important renal outcomes, such as persistent worsening of renal function, new onset proteinuria and progression to end-stage renal failure (ESRF), may to some degree circumvent any spurious relationship that is seen with MACE correlates.

Despite the lack of evidence supporting any direct causality effect on MACE, the onset of CIN following cardiac procedures remains an ominous event which should prompt additional clinical vigilance and early intervention.

## Proposed mechanisms underlying CIN

Intravascular CM are concentrated tri-iodinated benzene compounds that are radio-opaque as a result of their associated iodine moieties. All CM agents are cytotoxic and this may be compounded by the ionic strength, osmolality or viscosity of each specific agent.^31^ Ionic ‘hyper-osmolar’ solutions were the first to be used; however, these agents were found to be highly nephrotoxic and are now rarely administered.^32^ As such safer agents, including non-ionic ‘low-osmolar’ (LOCM) or ‘iso-osmolar’ (IOCM) solutions were developed. These formulations are significantly more viscous than blood plasma, with viscosity inversely related to osmolality^33^ (table 3). These two physicochemical properties of CM are thought to be implicated in the pathogenesis of CIN in addition to direct vasoactive and cytotoxic effects.^34^

**Table 3:** Comparison of CM agents by osmolality and viscosity

	Blood plasma	Iso-osmolar eg, Visipaque	Low-osmolar eg, Omnipaque	High-osmolar eg, Hypaque
Osmolality	290 mosmol/L	290 mosmol/L	890 mosmol/L	2100 mosmol/L
Viscosity	3–4 mPa s	8.8 mPa s	6.8 m mPa s	4.1 mPa s
CIN risk	N/A	Low	Low	High

CIN, contrast-induced nephropathy; CM, contrast media.

The kidney is particularly vulnerable to ischaemic injury as it is subjected to high metabolic and osmotic stress and it is supplied by an intricate microvascular circulation susceptible to local and systemic hypoperfusion. This is most evident in the outer medullary region of the kidney where oxygen requirements are high due to active sodium resorption in the ascending loop of Henle and the partial pressure of oxygen is low at approximately 20 mmHg.^35^ This relative ischaemia is related to the delicate blood supply provided for by the descending vasa recta (DVR), which is a long and narrow diameter vessel with high vascular resistance, and which may be further compounded by arteriovenous shunting.^36^ Patients with CKD are at an additional risk of renal ischaemia due to the increased metabolic demands placed on a reduced nephron bed which is often coupled with a compromised microvascular and macrovascular circulation.^37^

Although understanding of the complex pathogenesis of CIN is incomplete, the primary model identifies ischaemia in the vulnerable outer medullary region of the kidney as being pivotal.^36^ Following intravascular administration of CM, a prolonged period of renal vasoconstriction occurs due to an imbalance of local vasoactive mediators, such as nitrous oxide,^38^ adenosine, endothelin,^39^ prostaglandin and reactive oxygen species (ROS)^40^ which are released by the vascular endothelium in direct response to CM cytotoxicity. The resulting ischaemic tissue releases further noxious vasoactive mediators including ROS, thus prolonging the duration of vasoconstriction.

**Figure 1:**
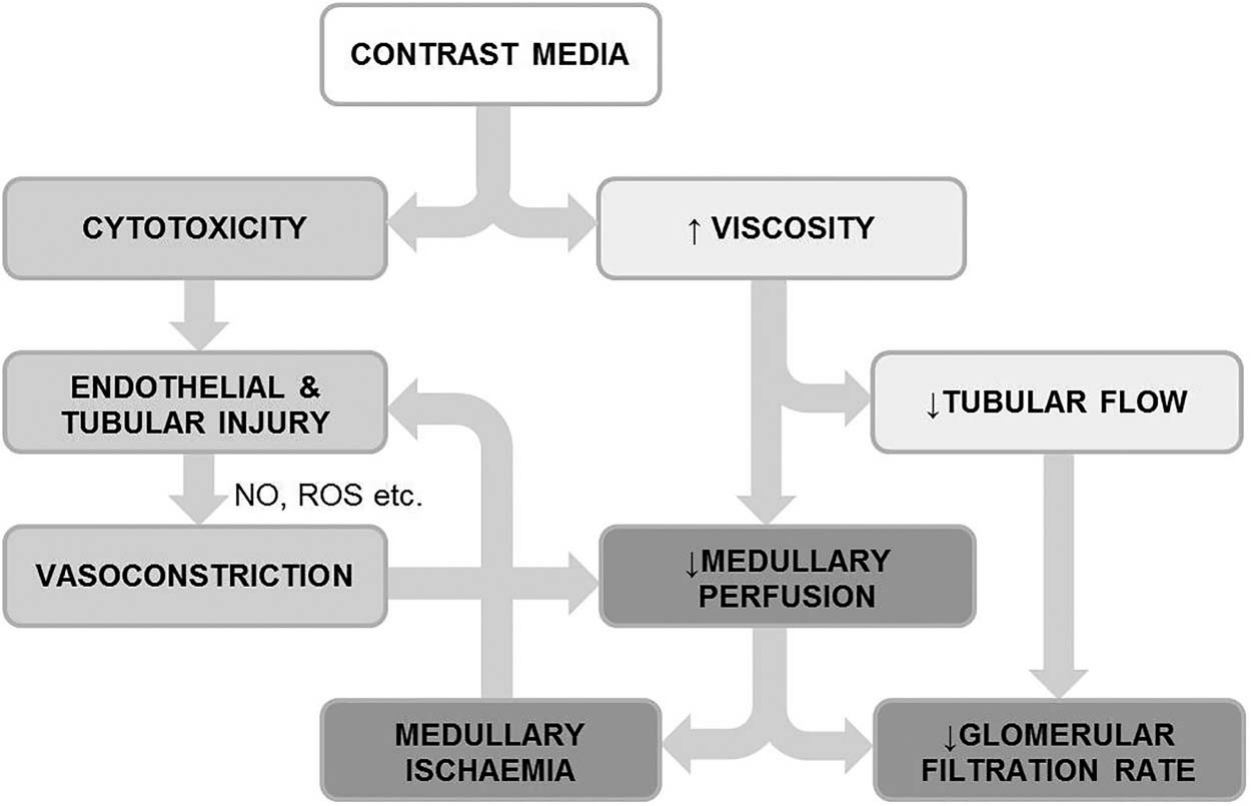
Pathophysiological mechanism underlying CIN is shown. CIN, contrast-induced nephropathy; NO, nitric oxide, ROS, reactive oxygen species. Adapted from Seeliger *et al*.^89^

The increased viscosity of the admixture of CM and blood plasma within the DVR results in a further reduction in medullary blood flow^41^ and the hyperosmolality of CM in plasma has been shown to cause red cell distortion and aggregation which may also contribute to reduced perfusion due to capillary obstruction.^42^ As CM is filtered and concentrated within the tubules, the resulting increase in viscosity causes tubular obstruction which, coupled with ROS release, induces an acute tubular injury.^43^ Thus, the combination of cytotoxicity, vasoconstriction and viscosity present a potent combination for the induction of medullary ischaemia/reperfusion injury (figure 1).

The risk of CIN is increased in elderly patients and those with diabetes or CKD, which may be due to the presence of endothelial dysfunction and an exaggerated vasoconstrictive response to CM.^44^ Equally, patients with poor pre-renal perfusion, such as those with congestive cardiac failure (CCF), renovascular disease or intravascular volume depletion, are also at increased risk of CIN due to the deleterious effect of renal vasoconstriction coupled with low preload. As observed in clinical studies, the presence of anaemia and thus reduced oxygen carrying capacity of blood would be expected to worsen ischaemia in the outer medullary region of the kidney.^45^

## Risk factors and risk assessment

In order to reduce the chance of CIN occurrence it is important to review the risk factors and indications for CM administration prior to any CM procedure. Most CIN risk factors can be assessed from the clinical history, physical examination and common laboratory investigations. Further risk factors may become apparent periprocedurally.

Pre-existent CKD is probably the most important pre-procedural risk factor for CIN. The European Society of Urogenital Radiology Consensus Working Panel^46^ in 1999 stated that CIN risk becomes clinically significant when baseline SCr concentration is ≥1.3 mg/dL (≥115 mmol/L) in men and ≥1.0 mg/dL (≥88.4 mmol/L) in women. These figures approximate to an eGFR <60 mL/min/1.73 m^2^, which defines CKD stages 3–5 and which is now generally recognised as the threshold for CIN risk.^47^ This simple biochemical assessment is the most widely adopted screening tool; however, it is important to recognise that SCr is an insensitive measure of renal function and that other risk factors are highly contributory to CIN, which can occur in patients without pre-existent CKD.^5^

Based on clinical experience, dehydration is a major risk factor for CIN; however, as it is largely a clinical diagnosis and is challenging to quantify, it has never been formally investigated in clinical trials. Hypotension, defined as a systolic blood pressure of <80 mm Hg for more than 60 min, is a recognised risk factor for CIN, attributable to intravascular volume depletion (eg, severe dehydration, haemorrhage or sepsis), cardiogenic shock (eg, AMI) or excessive vasodilation (eg, anaphylaxis) which results in renal hypoperfusion and thus increased sensitivity to CM-induced renal ischaemia. The presence of CCF classified according to New York Heart Failure Association III or IV, a recent history of pulmonary oedema,^9^ AMI,^6^ or LV ejection fraction of less than 45% have all been shown to be independent risk factors.^48^ ^49^ Diabetes mellitus is also an independent CIN risk factor as demonstrated in a number of clinical trials,^9^ ^48^ especially so when coexistent with CKD.^50^ Advanced age, usually quantified as over 75 years,^9^ is also associated with CIN. Anaemia is another important risk factor, usually defined as a haematocrit (HCT) of less than 0.39 in males or 0.36 in females.^45^ The coadministration of nephrotoxic agents (see table 6), are thought to increase the risk of CIN, although this is not well documented in clinical studies.^51^ There is conflicting evidence regarding the risk of concurrent treatment with ACE inhibitors and angiotensin receptor blocker’s (ARB's), however if part of established therapy, continuation is considered safer than the risk of withdrawal.^47^

Procedural factors such as the total volume of CM^9^ (>350 mL or >4 mL/kg) and previous CM exposure within 72 h^8^ are directly related to the development of CIN. A specific method for quantifying the maximum safe volume of contrast has been proposed by Laskey *et al*^52^ who demonstrated that a ratio of the volume of contrast media to creatinine clearance (V/CrCl) greater than 3.7:1 correlates strongly with the risk of developing CIN in patients with moderate CKD undergoing CA. In addition, the presence of periprocedural haemodynamic instability requiring the use of inotropic agents or intra-arterial balloon pump^9^ therapy is particularly high-risk feature. A number of these risk factors have been integrated into a well-known post-procedure risk scoring system and validated in a large cohort study by Mehran *et al*^9^ (table 4).

**Table 4:** The Mehran risk score for the prediction of CIN^9^

Mehran score periprocedural CIN risk factor				Score
Hypotension (SBP <80 mm Hg or >1 h of inotropic support)				5
Intra-arterial balloon pump therapy				5
Chronic heart failure, (NYHA III/IV or recent pulmonary oedema)				5
Age >75 years				4
Diabetes mellitus				3
Anaemia (male: HCT<0.39, female: HCT<0.36)				3
Estimated glomerular filtration rate <20 mL/min				6
Estimated glomerular filtration rate 20–40 mL/min				4
Estimated glomerular filtration rate 40–60 mL/min				2
Contrast media volume				1 per cc
**Score**	**<5**	**6–10**	**11–16**	**>16**
CIN risk	Low 7.5%	Moderate 14%	High 26.1%	Very high 57.3%
Dialysis risk	0.04%	0.12%	1.09%	12.6%

CIN, contrast-induced nephropathy; HCT, haematocrit; NYHA, New York Heart Failure Association; SBP, systolic blood pressure.

A similar scoring system has also been proposed by Tziakas *et al*^53^ who found that pre-existing renal disease, metformin use, history of previous PCI, peripheral arterial disease and ≥300 mL of contrast volume were also independent predictors of CIN. A limitation of these scoring systems is that calculation is only possible after CM has been administered. However, it is clinically desirable to be able to predict the risk of CIN before the patient is exposed to CM allowing appropriate precautionary measures to be taken. Such a pre-procedural CIN risk score has been proposed by Maioli *et al*,^8^ following validation in a prospective cohort study (table 5).

**Table 5:** A pre-procedural risk score for CIN (adapted from Maioli *et al*^8^)

Pre-procedural risk factor				Score
Prior CM exposure within 72 h				3
Left ventricular ejection fraction <45%				2
Pre-procedure SCr >baseline SCr				2
Baseline SCr >1.5 mg/dL				2
Diabetes mellitus				2
Creatinine clearance (eGFR) <44 mL/min				2
Age >73 years				1
**Score**	**0–3**	**4–6**	**7–8**	**>9**
CIN risk	Low 1.1%	Moderate 7.5%	High 22.3%	Very high 52.1%

CIN, contrast-induced nephropathy; CM, contrast media; eGFR, estimated glomerular filtration rate; SCr, serum creatinine.

A number of other novel CIN risk factors have been identified, including pre-procedure glucose levels^54^ ^55^ and low-density lipoprotein cholesterol;^56^ however, these have yet to be integrated into risk scoring systems. It may be possible to use commonly used cardiovascular risk scoring methods to approximate CIN risk, for example, a Global Registry of Acute Coronary Events score of >140 in patients with AMI having normal baseline renal function has been shown to predict risk of CIN in a small cohort study.^57^ A novel fluid status assessment method using Bio-Impedance Vector Analysis has also been demonstrated to independently predict CIN^58^ in a small clinical trial; however, it has not yet been translated into a CIN risk scoring system or guided volume repletion strategy.

## Established preventative measures

**Figure 2:**
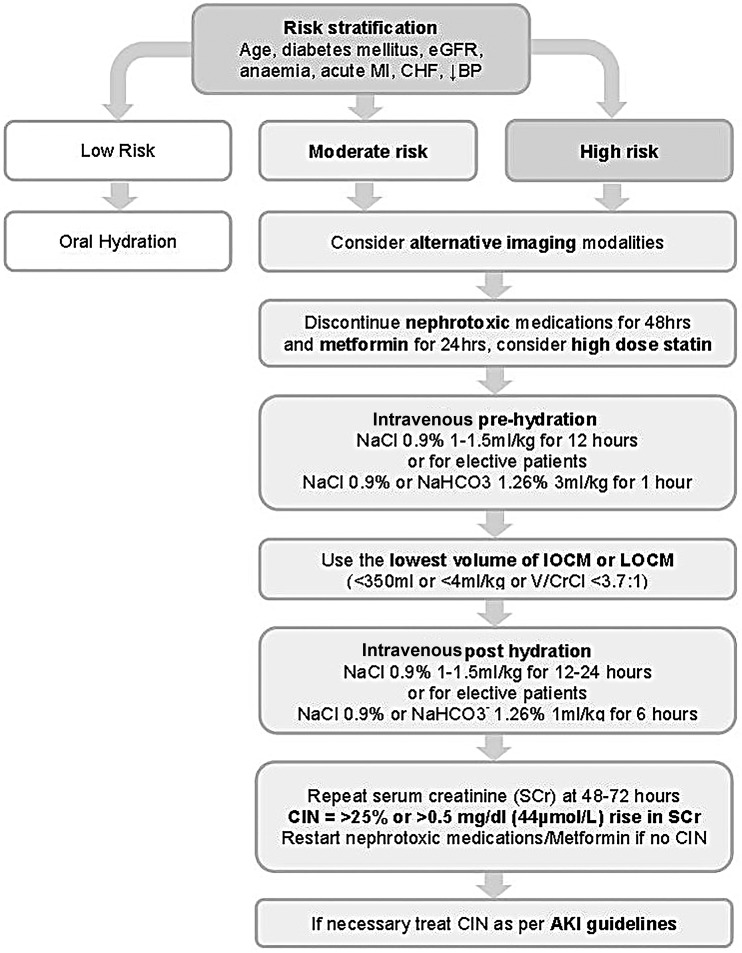
Algorithm for the prevention of CIN is shown. AKI, acute kidney injury; BP, blood pressure; CHF, chronic heart failure; CIN, contrast-induced nephropathy; eGFR, estimated glomerular filtration rate; IOCM, iso-osmolar contrast medium; LOCM, low-osmolar contrast medium; MI, myocardial infarction; NaCl, sodium chloride; NaHCO_3_^−^, sodium bicarbonate; Scr, serum creatinine; V/CrCl, volume of contrast media to creatinine clearance.

In 2014, the European Society of Cardiology published updated guidelines on CIN^59^ prevention which provides a framework for the use of the following evidence-based strategies (figure 2 and table 6). For all patients referred for CM procedures, a CIN risk assessment should be performed which includes baseline measurement of SCr and calculation of eGFR using a suitable formula, for example, Modification of Diet in Renal Disease or Chronic Kidney Disease–Epidemiology. If patients are identified as being at risk of CIN, particularly if eGFR is <40 mL/min, clinical indications for the CM procedure should be reviewed and preventative measures instigated. A single invasive approach should ideally be adopted, with CA followed by ad hoc PCI to reduce the risk of atheroembolic complications while minimising contrast volumes to <4 mL/kg or V/CrCl <3.7:1. However if a second CM procedure is necessitated, it is advisable to delay until adequate clearance of CM and recovery from any renal injury has occurred, which may be up to 2 weeks or as long as is clinically acceptable. It should be acknowledged that due to the overlap of common risk factors, patients at risk for CIN are also at significantly higher risk of cardiovascular events and therefore should not be excluded or unnecessarily delayed from receiving prognostic CM procedures unless the risks are considered to be excessive.

**Table 6:** European Society of Cardiology CIN prevention guidelines, 2014

Recommendation	Detail	Class	Level
Intravenous hydration with isotonic saline is recommended		I	A
Use of either LOCM or IOCM is recommended	<350 mL or <4 mL/kg or V/CrCl <3.7:1	I	A
IOCM use should be considered over LOCM		IIa	A
Short term, high-dose statin therapy should be considered	Rosuvastatin 20/40 mg or atorvastatin 80 mg or simvastatin 80 mg	IIa	A
Volume of CM should be minimised		IIa	B
A CIN risk assessment should be performed		IIa	C
In patients at very high CIN risk or when prophylactic hydration is impossible, furosemide with matched hydration may be considered over standard hydration	250 mL 0.9% saline intravenously over 30 min (or ≤150 mL in LV dysfunction) with 0.25–0.5 mg/kg of furosemide intravenous bolus. Adjust intravenous fluid rate to match urine output until >300 mL/h then perform CM procedure. Continue matched fluid replacement for 4 h post procedure	IIb	A
In severe CKD, prophylactic haemofiltration prior to complex PCI may be considered	Fluid replacement rate 1 L/h without negative loss, 0.9% sodium chloride intravenous hydration for 24 h post procedure	IIb	B
*N*-acetyl-cysteine instead of intravenous hydration is not recommended		III	A
Infusion of 8.4% sodium bicarbonate instead of 0.9% sodium chloride is not recommended		III	A
In severe CKD prophylactic renal replacement therapy is not routinely recommended		III	B

CIN, contrast-induced nephropathy; CKD, chronic kidney disease; CM, contrast medium; IOCM, iso-osmolar contrast medium; LOCM, low-osmolar contrast medium; LV, left ventricular; PCI, percutaneous coronary intervention; V/CrCl, volume of contrast media to creatinine clearance.

Adapted from Windecker *et al*.^59^

Patients should be advised to stop all non-essential nephrotoxic medications (table 7) for 24 h prior to and for 48 h following the CM procedure pending SCr measurement. It is also recommended that patients receiving intra-arterial CM with an eGFR of <60 mL/min/1.73 m^2^, or those receiving intravenous CM with an eGFR of <45 mL/min/1.73 m^2^, discontinue metformin for 48 h prior to CM exposure and restart once a 48 h SCr measurement excludes CIN. This is to mitigate the risk of lactic acidosis due to reduced renal clearance of metformin that may occur following a potential CIN episode, rather than metformin nephrotoxicity per se.

**Table 7:** Nephrotoxic medications requiring withdrawal 24 h pre-procedure

Drug class	Examples
Non-steroidal anti-inflammatory	Naproxen, Ibuprofen, Diclofenac, Celecoxib^90^
Antibiotics	Aminoglycosides: (Gentamycin, Tobramycin, Amikacin)^91^
Antifungals	Amphotericin B^92^
Antivirals	Acyclovir, Tenofovir, Foscarnet^93^
Immunomodulatory	Ciclosporin A^94^
Antineoplastic	Cisplatin, Ifosfamide, Mitomycin^95^

Recent studies have investigated whether IOCM formulations, which are thought to induce less osmotic stress despite generally having higher viscosity, are preferable over LOCM. A number of meta-analyses have been performed, some of which suggest the superiority of IOCM; however, others have shown no benefit.^60^ ^61^ The current guidelines recommend the use of either IOCM or LOCM, although a preference for IOCM is reasonable, with the more important proviso that the minimum amount of CM required for diagnostic accuracy is used. During CA or PCI, the use of biplane imaging by experienced operators may reduce the amount of contrast required as simultaneous orthogonal views can be acquired following each CM injection.^62^

The most effective prophylactic intervention is provision of adequate hydration prior to CM exposure. Supplementing intravascular volume ensures renal blood flow is maintained and acts to dilute CM in both blood plasma and tubular filtrate. In lower risk ambulant patients, the oral route may be appropriate if adequate fluid intake is assured. However, in moderate/higher risk or in hospitalised patients intravenous hydration with a crystalloid fluid is preferred over oral hydration as it guarantees delivery of appropriate fluid volumes and has been demonstrated as superior in clinical trials.^63^ The choice of which crystalloid to use is, however, less clear; when compared with isotonic (normal) saline (0.9%), intravenous sodium bicarbonate (1.26%) may have additional ROS scavenging properties mediated through urine alkalinisation^64^ and lacks chloride ions that are thought to exacerbate renal vasoconstriction.^65^ Two recent meta-analyses have demonstrated a modest reduction in CIN when using intravenous sodium bicarbonate 1.26% as compared with isotonic saline,^66^ ^67^ although no significant mortality benefit has been demonstrated. In view of the current lack of evidence supporting the use of sodium bicarbonate 1.26% and with some studies offering conflicting evidence,^68^ the current ESC guidelines recommend pre-hydration with sodium chloride 0.9% at 1–1.5 mL/kg/h for 12 h pre-procedure and up to 24 h post procedure. For elective day case patients and for those with CCF in whom large volumes of intravenous fluid may provoke pulmonary oedema,^69^ it is also reasonable to use an alternative protocol delivering a shorter duration and volume of sodium chloride 0.9% (table 8).

**Table 8:** Intravenous pre-hydration regimes, Updated ESUR guidelines 2011^47^

Intravenous fluid	Pre-hydration	Post-hydration
Isotonic saline (0.9%)	12 h, 1–1.5 mL/kg/h	12–24 h, 1–1.5 mL/kg/h
Isotonic saline (0.9%) or sodium bicarbonate (1.26%)	1 h at 3 mL/kg/h	6 h at 1 mL/kg/h

ESUR, European Society of Urogenital Radiology.

All patients determined as being at risk of CIN should have SCr levels measured between 48 and 72 h following CM exposure. If CIN is diagnosed, then it should be managed using recommended AKI guidelines, such as the recent European Best Practice position statement on AKI.^70^ This includes follow-up SCr measurements, withdrawal of nephrotoxic medications and unnecessary loop diuretics, electrolyte and hydration optimisation, nutritional advice and, if severe AKI occurs, early hospitalisation with referral to a specialist nephrologist.

## Novel preventative measures

A number of prophylactic pharmacological agents have been investigated; however, at present the evidence for benefit in CIN prevention is limited. Originally one of the most promising agents, *N*-acetyl-cysteine (NAC), is inexpensive, well tolerated and has both antioxidant and vasodilatory properties. Several large RCTs have shown oral NAC at dose of 600 mg twice a day for 24 h pre-procedure and post procedure reduces the incidence of CIN.^71^ ^72^ However meta-analyses^73^ ^74^ have failed to reach consensus, most likely due to clinical heterogeneity, variable reporting and publication bias in the included studies.^75^ As such the ESC guidelines recommend that NAC is not to be used alone, although it may be used in addition to standard intravenous hydration regimes.^70^ More recently high-dose statin therapy (eg, rosuvastatin 40/20 mg, atorvastatin 80 mg or simvastatin 80 mg) has shown efficacy in preventing CIN in statin-naïve patients in several clinical studies^76^ ^77^ and as such is regarded as reasonable preventative therapy in the current ESC guidelines. Other pharmaceutical agents with antioxidant (eg, ascorbic acid) and vasodilatory properties have also been investigated and although some have shown promise, further evaluation is required (table 9).

**Table 9:** Potential pharmacological prophylactic agents

Drug name	Study	Outcome: treatment vs control
High-dose statins	PRATO-ACS study^76^ n=504 Marenzi *et al*^77^ n=1134	CIN 6.7% vs 15.1% OR 0.38; 95% CI 0.20 to 0.71; p=0.003 CIN 5.5% vs 15% RR=0.37; 95% CI 0.25 to 0.55; p<0.0001
*N*-acetyl cysteine	Gonzales *et al*^73^ Meta-analysis n=2746	High-quality RCTs—no CIN benefit RR=0.87; 95% CI 0.68 to 1.12, p=0.28 Low-quality RCTs—high CIN benefit RR=0.15; 95% CI 0.07 to 0.33, p<0.0001
Ascorbic acid	Spargias *et al*,^96^ RCT n=231	CIN 9% vs 20% OR 0.38 95% CI 0.17 to 0.85; P=0.02
Theophylline	Ix *et al*^97^ Meta-analysis, n=480	Difference in mean SCr 11.5 µmol/L 95% CI 5.3 to 19.4 µmol/L, p=0.004
Iloprost	Spargias *et al*^98^ RCT n=208	CIN 8% vs 20% OR 0.29 95% CI 0.12 to 0.69; p=0.005
Prostaglandin E1	Li *et al*^99^ n=163, RCT	CIN 3.7 vs 11.1% p<0.05
Trimetazidine	Shehata *et al*,^100^ RCT n=100	CIN 12% vs 28% (p<0.05) (lower Troponin-T in Trimetazidine group)
Atrial natriuretic peptide	Morikawa *et al*,^101^ RCT n=254	CIN 3.2% vs 11.7% OR 0.24; p=0.016

Conflicting or negative evidence.

Fenoldopam,^102^ dopamine,^103^ calcium channel blockers,^104^ L-arginine,^105^ furosemide without matched

hydration,^106^ mannitol,^107^ endothelin receptor antagonists.^108^

CIN, contrast-induced nephropathy; RCT, randomised controlled trial; RR, relative risk; SCr, serum creatinine.

There has also been considerable interest in novel interventional therapies that may offer additional protection when combined with conventional therapy. These can be characterised into three categories: direct renal protection, hydration optimisation and CM delivery and extraction technologies. The prototypical renal protection approach is exemplified by remote ischaemic preconditioning, which has been shown in many models to be cytoprotective against ischaemia/reperfusion injury, believed to be characteristic of CIN.^78^ Several small RCTs have shown promise using cycles of brief, non-injurious remote tissue ischaemia to trigger renal protection. Both blood pressure cuff inflation on the upper arm prior to CM exposure (preconditioning)^79^ and catheter balloon inflation in the target coronary artery following PCI (post-conditioning)^80^ ^81^ have demonstrated a 60%–70% reduction in the incidence of CIN. Although encouraging, larger Phase II/III studies are required to confirm efficacy of this safe and cost–effective intervention.

Two hydration optimisation strategies have been trialled in CIN: LV end diastolic pressure (LVEDP) guided volume expansion and high urine output matched fluid replacement (RENALGUARD). The optimum rate and volume of intravenous fluid delivery represents a significant challenge: under-hydration increases CIN risk, whereas overhydration may precipitate acute pulmonary oedema in vulnerable patients with severe CKD and CCF. The recent POSEIDON RCT^82^ demonstrated that LVEDP-guided volume expansion in at-risk patients was safe and significantly reduced the incidence of CIN from 16.3% (28/172) in controls to 6.7% (12/178) in the fluid-guided group. An alternative fluid management system is delivered by the RENALGUARD system, which maintains a high urine output (>300 mL/h) using balanced intravenous isotonic saline (0.9%) delivery and intravenous furosemide infusion (0.25 mg/kg). The REMEDIAL II study^83^ demonstrated superiority of the RENALGUARD system plus oral NAC in preventing CIN (11%, 16/146) against a control group receiving sodium bicarbonate (1.26%) regimen plus oral NAC (20.5%, 30/146; OR 0.47; 95% CI 0.24 to 0.92). As such these novel therapies hold promise for higher risk patients especially those who are physiologically unable to tolerate large intravenous fluid volumes.

In order to minimise CM load, novel automated contrast injection devices have been developed which decrease the volume of CM used and which have been shown to reduce the incidence of CIN.^84^ It has been proposed that rapid removal of CM from the blood pool may have benefit in preventing CIN; although prophylactic HD has not been shown attenuate the incidence of CIN,^85^ some benefit has been observed with both pre-procedural and post-procedural^86^ haemofiltration (HF) and simultaneous HF,^87^ which may be partially explained through optimisation of periprocedural intravascular volumes. However, HF is a resource-intensive therapy that should be reserved for very-high-risk patients, such as for those with pre-dialysis ESRF^88^ or those with severe CKD undergoing complex PCI. Direct extraction of CM in coronary venous blood, captured using a coronary sinus catheter, is an interesting experimental intervention^89^ which has yet to be proven in clinical trials.

## Summary and conclusion

CIN represents a significant clinical and health economic problem that may be under-recognised through limitations in the currently available biomarkers. Although often a transient injury, CIN may progress to significant persistent renal impairment, ESRF and adverse cardiovascular outcomes. There are a number of recognised risk factors, although the prediction of CIN, particularly prior to contrast administration, remains challenging. Current interventions are largely centred on the avoidance of dehydration, the withdrawal of nephrotoxic agents and minimisation of contrast load, which has limited efficacy in preventing CIN in vulnerable patients. The unmet clinical need in CIN therefore resides in accurate prediction, effective intervention and rapid detection to prevent adverse cardiorenal outcomes. Each of these areas, particularly predictive risk scoring systems, innovative pharmacological and mechanical interventions and novel biomarkers are currently the subject of intensive research and development that may lead to the future development effective strategies to mitigate the risk of CIN.

**You can get CPD/CME credits for Education in Heart**

Education in Heart articles are accredited by both the UK Royal College of Physicians (London) and the European Board for Accreditation in Cardiology—you need to answer the accompanying multiple choice questions (MCQs). To access the questions, click on **BMJ Learning: Take this module on BMJ Learning** from the content box at the top right and bottom left of the online article. For more information please go to: http://heart.bmj.com/misc/education.dtl

- **RCP credits:** Log your activity in your CPD diary online (http://www.rcplondon.ac.uk/members/CPDdiary/index.asp)—pass mark is 80%.
- **EBAC credits:** Print out and retain the BMJ Learning certificate once you have completed the MCQs—pass mark is 60%. EBAC/ EACCME Credits can now be converted to AMA PRA Category 1 CME Credits and are recognised by all National Accreditation Authorities in Europe (http://www.ebac-cme.org/newsite/?hit=men02).

**Please note:** The MCQs are hosted on BMJ Learning—the best available learning website for medical professionals from the BMJ Group. If prompted, subscribers must sign into *Heart* with their journal's username and password. All users must also complete a one-time registration on BMJ Learning and subsequently log in (with a BMJ Learning username and password) on every visit.
